# Initial Investigations of Intrinsically Disordered Regions in Inherited Retinal Diseases

**DOI:** 10.3390/ijms24021060

**Published:** 2023-01-05

**Authors:** Karen E. Lee, Rebecca Procopio, Jose S. Pulido, Kammi B. Gunton

**Affiliations:** 1 Pediatric Ophthalmology & Adult Strabismus Service, Wills Eye Hospital, 840 Walnut Street, Philadelphia, PA 19107, USA; 2 Retina Service, Wills Eye Hospital and Mid Atlantic Retina, 840 Walnut Street, Philadelphia, PA 19107, USA; 3 Department of Translational Ophthalmology, Wills Eye Hospital, 840 Walnut Street, Philadelphia, PA 19107, USA

**Keywords:** inherited retinal diseases, intrinsically disordered proteins, missense variants, protein structure

## Abstract

Intrinsically disordered regions (IDRs) are protein regions that are unable to fold into stable tertiary structures, enabling their involvement in key signaling and regulatory functions via dynamic interactions with diverse binding partners. An understanding of IDRs and their association with biological function may help elucidate the pathogenesis of inherited retinal diseases (IRDs). The main focus of this work was to investigate the degree of disorder in 14 proteins implicated in IRDs and their relationship with the number of pathogenic missense variants. Metapredict, an accurate, high-performance predictor that reproduces consensus disorder scores, was used to probe the degree of disorder as a function of the amino acid sequence. Publicly available data on gnomAD and ClinVar was used to analyze the number of pathogenic missense variants. We show that proteins with an over-representation of missense variation exhibit a high degree of disorder, and proteins with a high amount of disorder tolerate a higher degree of missense variation. These proteins also exhibit a lower amount of pathogenic missense variants with respect to total missense variants. These data suggest that protein function may be related to the overall level of disorder and could be used to refine variant interpretation in IRDs.

## 1. Introduction

Our understanding of protein function is dependent on crystallography, biochemical studies, and computational analysis of protein structure. This structure–function paradigm has been widely applied to proteins that have a well-defined secondary and tertiary structure [1]. Some protein sequences, which lack hydrophobic amino acids and therefore do not form a hydrophobic core, do not form a three-dimensional structure [2]. These regions are referred to as intrinsically disordered regions (IDRs) [1]. Certain amino acids are more prone to promote IDRs (proline, glutamic acid, serine, glutamine, lysine, alanine, and glycine), while other amino acids are more prone to promote ordered regions (aspartic acid, threonine, and arginine). Some are not causative for either disorder or order and are considered neutral (methionine, asparagine, valine, histidine, leucine, phenylalanine, isoleucine, tyrosine, tryptophan, and cysteine) [3,4]. Proteins made up entirely of IDRs are referred to as intrinsically disordered proteins (IDPs). These proteins and protein regions fluctuate on a continuum of conformations, dynamically shifting in a way that is functional for their role within the cell [5,6]. Not only do IDRs regulate protein conformations, but there is evidence that human proteasomes with N-terminal IDRs have significantly shorter half-lives than proteasomes without these features [7]. Primary sequences of IDRs may include molecular recognition features or short linear motifs [8]. Molecular recognition features are short 10–70 amino acid motifs that facilitate protein interaction with other smaller molecules, and short linear motifs are conserved sequences of less than ten amino acids that mediate protein–protein interactions involved in cell signaling [8].

Recently, studies of IDRs and IDPs have become increasingly relevant due to their involvement in important cellular processes, including cell signaling, protein regulation, and transmembrane transport [9]. The disorder allows these proteins to change conformation, allowing flexibility [10]. Because of their importance in cellular interaction, IDPs are tightly regulated and, when altered, are implicated in various diseases [9]. More specifically, IDPs have been analyzed in the context of cancer, cardiovascular, and neurodegenerative disease and are postulated as a target for therapeutic modalities [10,11,12].

The role of IDPs and proteins with IDR regions has yet to be examined in the context of inherited retinal disease (IRD). The inherited retinal disease includes a heterogeneous group of diseases that lead to visual impairment caused by numerous nuclear and mitochondrial genetic aberrations [13]. Recently, Tanner et al., demonstrated that six IRD genes (*SAMD11*, *ALMS1*, *WFS1*, *RP1L1*, *KCNV2*, *ADAMTS18*) exhibit an overrepresentation of nonsynonymous missense variant [14] and that these may constitute “noisy genes,” which should be interpreted with caution when found in patients. Though our understanding of why certain proteins tolerate higher percentages of missense variation is currently limited, we hypothesized that these proteins have a higher percentage of IDRs and thus allow more flexibility in conformations. In this study, we evaluated 14 genes implicated in IRDs by examining the predicted ordered and disordered regions within each protein. We categorized proteins into four groups: (1) proteins with overrepresentation of missense variants (SAMD11, ALMS1, WFS1, RP1L1, KCNV2, and ADAMTS18), (2) transmembrane transport (CNGB1, CNGA1, TRPM1, ABCA4, BEST1, and KCNV2), (3) internal or structural proteins of the photoreceptors that are essential in visual function (RHO and RPE65), and (4) secreted proteins (TIMP3 and ADAMTS18). We investigated the disorder content in each of these proteins and examined their relationship with the number of nonsynonymous missense variants.

## 2. Results

### 2.1. Analysis of Degree of Disorder

Group 1 proteins, namely those that previously had been shown to have an over-representation of nonsynonymous variants, exhibited the highest degree of disorder with content found to be 96.5, 87.7, 80.4, 25.3, 22.8, and 13.6% for ALMS1, RP1L1, SAMD11, KCNV2, WFS1, and ADAMTS18, respectively. Metapredict analysis graphs for ALMS1 and RHO are shown in Figure 1 and Figure 2, respectively. The remaining proteins in Groups 1–4 are shown in Figures S1–S12.

**Figure 3:**
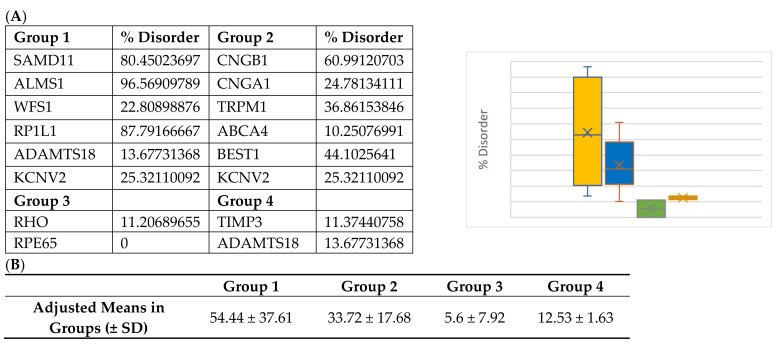
(**A**) Disorder content and box plot comparisons for proteins in Groups 1–4. Disorder content is listed as a percentage in the chart and decimal in the box plot. Yellow = Group 1: over-representation of missense variants; Blue = Group 2: transmembrane proteins; Green = Group 3: internal and structural proteins of photoreceptors; Red = Group 4: secreted proteins. (**B**) Mean disorder in Groups 1–4.

Interestingly, Group 3 proteins (important internal proteins for structure and function in photoreceptors) exhibited the lowest degree of disordered regions. The intrinsic disorder was 11.4% for RHO and 0% for RPE65. On the other hand, Group 2 (transmembrane transport proteins expressed in the retina) would be expected to exhibit a high degree of disorder. In fact, we found disorder content of 60.9% for CNGB1, 44.1% for BEST1, 36.9% for TRPM1, 25.3% for KCNV2, 24.8% for CNGA1, and 10.2% for ABCA4. Finally, because ADAMTS18 is also a secreted protein, we expanded our analysis to include TIMP3, a protein that inhibits enzymes and degrades matrix components. Disorder content in terms of percentages and box plots is displayed in Figure 3A. The comparison of mean disorder content across the four groups is shown in Figure 3B. The ANOVA showed *p* = 0.129, likely because of the small number of proteins evaluated.

### 2.2. Proteins with Higher Disordered Regions Tolerate a Higher Amount of Total Missense Variation

**Figure 4:**
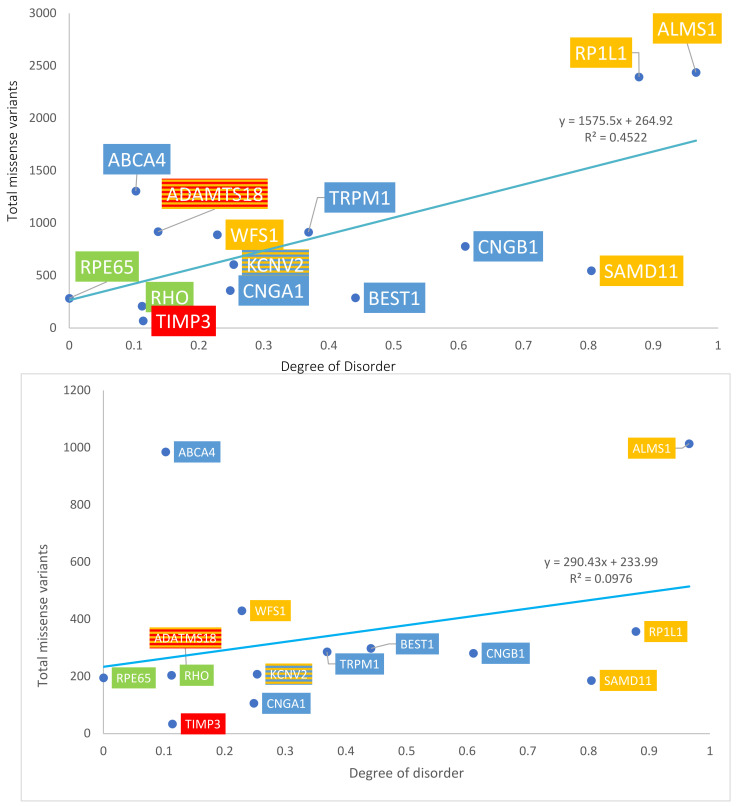
Graph of disorder content versus the total number of missense variants. Yellow = Group 1: over-representation of missense variants; Blue = Group 2: transmembrane proteins; Green = Group 3: internal and structural proteins of photoreceptors; Red = Group 4: secreted proteins. The number of total missense variants obtained from gnomAD data (**top** panel) and ClinVar data (**bottom** panel).

**Figure 5:**
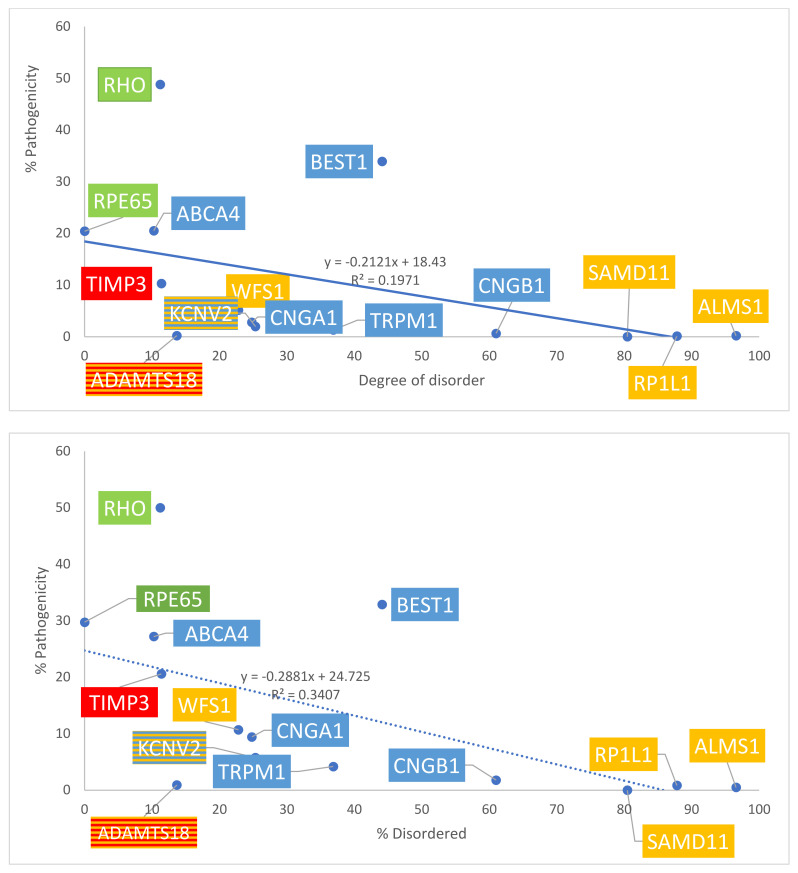
Graph of disorder content with respect to % pathogenicity. Percentages are graphed. Yellow = Group 1: over-representation of missense variants; Blue = Group 2: transmembrane proteins; Green = Group 3: internal and structural proteins of photoreceptors; Red = Group 4: secreted proteins. The number of total missense variants was obtained from gnomAD data (**top** panel) and ClinVar data (**bottom** panel).

The number of total missense variants for each protein in the four groups was determined using both ClinVar and gnomAD sources. Data are summarized in Table 1A. With gnomAD data, linear regression demonstrated a significant positive correlation between the degree of disorder and the total number of missense variants (*p* = 0.008, R^2^ = 0.45, R= 0.67). This is displayed in Figure 4, top panel. With ClinVar data, linear regression showed a similar positive linear trend, though the relationship did not approach significance (*p* = 0.277, R^2^ = 0.0976, R = 0.31). This is displayed in Figure 4, bottom panel. The ratio between the number of pathogenic missense variants and total missense variants was calculated for each protein to assess the percentage of pathogenic missense variants; results are summarized in Table 1B. This ratio is termed % pathogenicity. With ClinVar data, linear regression showed a significant relationship between the degree of disorder and % pathogenicity (*p* = 0.028, R^2^ = 0.34, R = 0.58) (Figure 5, bottom panel). A similar relationship was demonstrated with gnomAD data, though it did not approach statistical significance (*p* = 0.11, R^2^ = 0.197, R = 0.44) (Figure 5, top panel). Structural proteins exhibit the highest % pathogenicity, and over-representation of missense variants shows the lowest % pathogenicity. Prior to a Benjamini–Hochberg adjustment, *p* = 0.008 and 0.028 were considered significant. Following adjustment, *p* = 0.008 and 0.028 achieved significance.

## 3. Discussion

Proteins with comparatively higher amounts of the disorder have a higher tolerance for missense variation. This is because intrinsically disordered protein regions lack a stable 3D structure, which enables rich conformational adaptability and potential for efficient interaction with diverse binding targets. It also appears that IRD proteins with higher amounts of the disorder have an over-representation of missense variants and transmembrane proteins. Conversely, proteins that are very structured were noted to be those that are involved in visual function. This suggests that protein function may be related to the overall level of disorder and could be used to refine variant interpretation in IRDs. Previous studies have demonstrated that IDRs are more abundant in membrane proteins than in the complete proteome, with more than 50% of transmembrane proteins containing IDRs composed of at least 30 residues [15,16]. Our results show that Group 2 transmembrane proteins exhibit the second-highest degree of disorder, confirming this finding. Wang et al., have demonstrated the enrichment of missense mutations on protein interaction interfaces [17], underscoring the impact of missense variants on protein regulatory function. Furthermore, 20% of nonsynonymous SNVs are located in IDRs [18], suggesting that disease variants in IDRs are equally critical to those in structured domains. 

We showed a significant relationship between disorder content and the percentage of pathogenic missense variants as a fraction of total missense variants. The most ordered proteins are in Group 3 (structural and internal proteins that are essential for visual function) and exhibited the highest pathogenic missense variants to total missense variants ratio. This suggests that highly ordered proteins may be more susceptible to acquiring pathogenic missense variants, perhaps because of their comparatively limited ability to undergo conformational changes and thus maintain function. In support of our findings, Laddach et al., showed that pathogenic missense variants tended to localize to ordered regions [19]. Variants occurring in ordered regions may more greatly destabilize an overall protein compared with those occurring in a disordered region, which may explain why Group 3 exhibited the greatest number of pathogenic missense variants. Our initial studies are encouraging in showing a relationship between disorder and variant acceptance, and further studies are warranted. Present in silico mutation analyses only investigate the effect of amino acid substitutions on the surrounding local milieu without determining if the surrounding region is ordered or disordered; adding this assessment as well might improve our ability to determine the in silico determination of variant pathogenicity. 

There are several limitations to this study. Only a selected number of proteins implicated in IRD were probed, so follow-up studies analyzing a greater number of proteins with broad functional roles are needed for a better understanding of how disordered protein regions are implicated in IRDs. Only two proteins in Group 3 were evaluated in this initial study, and these are internal proteins of the photoreceptors that are also essential for visual function. Though we divided the proteins into four groups, the number of proteins was too small to obtain a statistically significant difference in disorder between the groups. It is important to note that this study represents the first evaluation of IDR in IRDs. For the presented proteins, we demonstrated a positive relationship between IDRs and total missense variants, and future studies may further evaluate this relationship in other proteins involved in other IRDs. We categorized proteins in regard to their importance in visual functionality, but whether certain proteins are more important than others is not within the scope of this study. Therefore, not all proteins perfectly follow the linear regression model. Studies on the natural selection of proteins and their association with IDRs will help elucidate the relative roles of essential proteins within the cell.

Another possible limitation is that Metapredict, an in silico evaluation of IDRs, may not be fully accurate in disorder prediction. However, Metapredict version 2 was shown to correctly classify both human transcription factors and de novo synthetic proteins [20], and the original version was demonstrated to have a low average error rate of two residues in 100 [21]. Additionally, the cutoff threshold used was 0.5, such that any residue above a disorder score of 0.5 was considered to be “disordered,” and below 0.5 was considered “ordered” [21]. This was an increase in threshold score from the legacy version and predicted a much larger percentage of proteins to be disordered than before, thus decreasing the number of false negatives [20]. Even with a high degree of accuracy, Uppal et al., recently identified a small IDR of 18 amino acids in RPE65 [22]. This suggests that in silico predictions may not fully resolve small-region IDRs. Furthermore, ClinVar is updated on a routine basis, and we used the January 2022 version. Newer versions may show minor changes when compared with our findings. There have been studies assessing various deep-learning algorithms that predict disordered protein regions [23,24]. Eventually, our analysis may become automated as part of artificial intelligence efforts. Finally, future studies examining how IDRs affect the efficacy of gene therapy are definitely warranted.

## 4. Materials and Methods

A variety of publicly available bioinformatics tools and databases were used in the analysis of 14 proteins categorized into four groups.

### 4.1. Prediction of Intrinsically Disordered Regions

The amino acid sequences of each protein used in this study were obtained through the National Institutes of Health (NIH) National Library of Medicine Protein search tool. The Mane Select transcript was selected for each protein. The sequences were then run through Metapredict to plot the intrinsically disordered regions (IDRs). Metapredict is an accurate, high-performance predictor of protein disorder and structure that reproduces consensus disorder scores [21]. Consensus scores report the percentage of independent disorder predictors that would predict a residue as disordered [21]. The accuracy and execution time of Metapredict has been previously compared to existing competitor predictors. Analysis showed that, while Metapredict was approximately two residues per 100 (less accurate than the highest-performing predictor), it took only 40 s to predict the entire testing dataset compared with approximately one month for the highest-performing predictor [21].

We input amino acid sequences for each protein into Metapredict and obtained graphs of disorder as a function of amino acid residue. To complement the graphical representation of disorder for each protein, we calculated the degree of disorder (defined as the percentage of IDRs) by taking the number of amino acids within the IDRs divided by the total number of amino acids.

### 4.2. Variant Pathogenicity

Databases gnomAD and ClinVar were used to examine the number of pathogenic missense mutations and total missense mutations for each protein. The gnomAD database is currently the largest and most widely-accessed publicly available reference population collection [25] that provides rapid variant analysis. ClinVar is maintained by the National Institutes of Health and is a public archive of human genetic variants [26]. ClinVar variants noted in the January 2022 version and included in gnomAD were used to examine the number of pathogenic missense mutations compared with total missense mutations for each protein. The number of pathogenic missense variants was obtained on gnomAD by selecting only “pathogenic/likely pathogenic” and “Missense/inframe indel” and manually excluding any inframe indel variants that may have been included. The number of total missense variants was obtained on gnomAD as the observed single-nucleotide variants (SNVs) for the missense category. For each representative protein, genetic maps of pathogenic missense mutations and total missense mutations were plotted with Metapredict.

### 4.3. Statistical Analysis

Linear regression was performed in Microsoft Excel to determine the degree of correlation between (1) disorder content and % pathogenicity and (2) disorder content and the total number of missense variants using both gnomAD and ClinVar data. The coefficient of determination (R^2^) was determined, and a *p*-value of <0.05 was considered significant.

The Benjamini–Hochberg adjustment was employed to decrease the false discovery rate for multiple hypotheses testing. Critical values were calculated for a total of four individual *p*-values with the formula (i/m)×Q, where i designates the *p*-value rank, m is the total number of *p*-values, and Q is the false discovery rate set at 20%.

## 5. Conclusions

Our study represents an initial explanatory investigation, and many conclusions are tentative. For these proteins, the hypothesis is valid. Future work will include categorizing each variant based on changes to disordered or ordered amino acids and localizing variants to protein regions. Furthermore, it would be important to elucidate which groups of proteins exhibit higher percentages of changes to disordered and ordered amino acids to better predict the relationship between genetic variants, protein function, and disease.

## Figures and Tables

**Figure 1:**
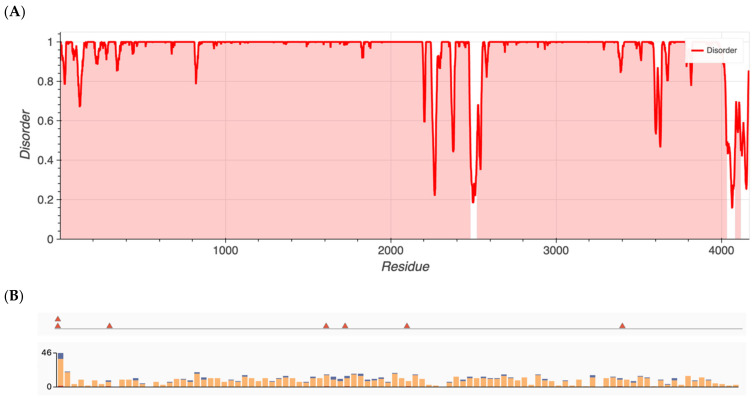
(**A**) Metapredict generated plots of intrinsically disordered regions as a function of amino acid residue for ALMS1, a protein with the highest amount of disorder. (**B**) Overlay of pathogenic missense variants (**top**) and total missense variants (**bottom**) for ALMS1 generated from ClinVar’s data release.

**Figure 2:**
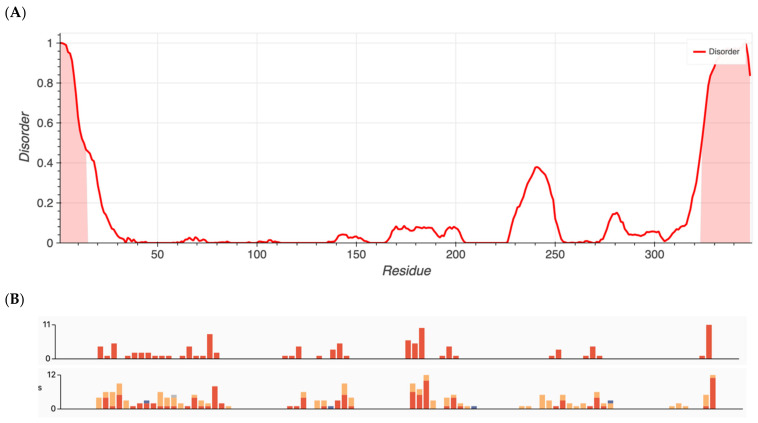
(**A**) Metapredict generated plots of intrinsically disordered regions as a function of amino acid residue for RHO, a protein with the lowest amount of disorder. (**B**) Overlay of pathogenic missense variants (**top**) and total missense variants (**bottom**) for RHO generated from ClinVar’s data release.

**Table 1:** (**A**) Number of total missense variants, as obtained from ClinVar and gnomAD data and pathogenic missense variants for proteins in Groups 1–4. (**B**) Percentage of pathogenic missense variants over total missense variants (% pathogenicity), as obtained from ClinVar and gnomAD data for proteins in Groups 1–4.

(A)			
Protein Name	# Total Missense Variants (Clinvar)	# Total Missense Variants (gnomAD)	# Pathogenic Missense Variants
SAMD11	186	546	0
ALMS1	1014	2437	5
WFS1	430	890	46
RP1L1	357	2394	3
ADAMTS18	220	919	2
CNGB1	281	779	5
KCNV2	208	606	12
CNGA1	106	358	10
TRPM1	286	914	12
ABCA4	985	1306	268
BEST1	298	289	98
RHO	204	209	102
RPE65	195	284	58
TIMP3	34	68	7
**(B)**			
**Protein Name**	**% Pathogenicity (Clinvar)**		**% Pathogenicity (gnomAD)**
SAMD11	0		0
ALMS1	0.493096647		0.205170291
WFS1	10.69767442		5.168539326
RP1L1	0.840336134		0.125313283
ADAMTS18	0.909090909		0.217627856
CNGB1	1.779359431		0.641848524
KCNV2	5.769230769		1.98019802
CNGA1	9.433962264		2.793296089
TRPM1	4.195804196		1.312910284
ABCA4	27.20812183		20.52067381
BEST1	32.88590604		33.9100346
RHO	50		48.80382775
RPE65	29.74358974		20.42253521
TIMP3	20.58823529		10.29411765

## Data Availability

Metapredict online (v2.1) can be found at: https://metapredict.net/ (accessed on 18 October 2022). The gnomAD database can be found at: https://gnomad.broadinstitute.org/ (accessed on 18 October 2022).

## Supplementary Materials

The following supporting information can be downloaded at: https://www.mdpi.com/article/10.3390/ijms24021060/s1.
