# Supplementary material for: Initial Investigations of Intrinsically Disordered Regions in Inherited Retinal Diseases
Source: Int J Mol Sci. 2023 Jan 5;24(2):1060. doi: 10.3390/ijms24021060 (PMC9861917; doi:10.3390/ijms24021060)
Supplement: Supplementary file 1 [file ijms-24-01060-s001.zip › ijms-2010834-supplementary.pdf]

Fig. S1a.

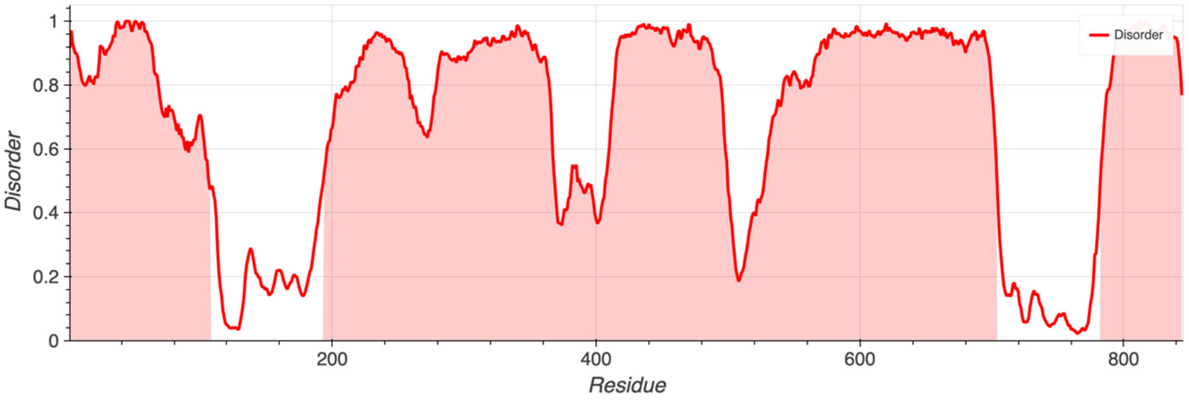

Fig. S1b.

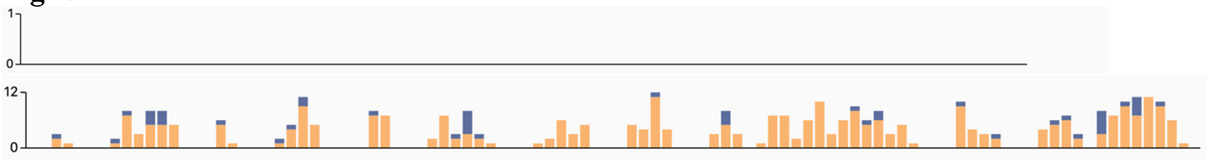

Figure S1. (a) Metapredict generated plots of intrinsically disordered regions as a function of amino acid residue **(b)** overlay of pathogenic missense variants (top panel) and total missense variants (bottom panel) for SAMD11.

Fig. S2a.

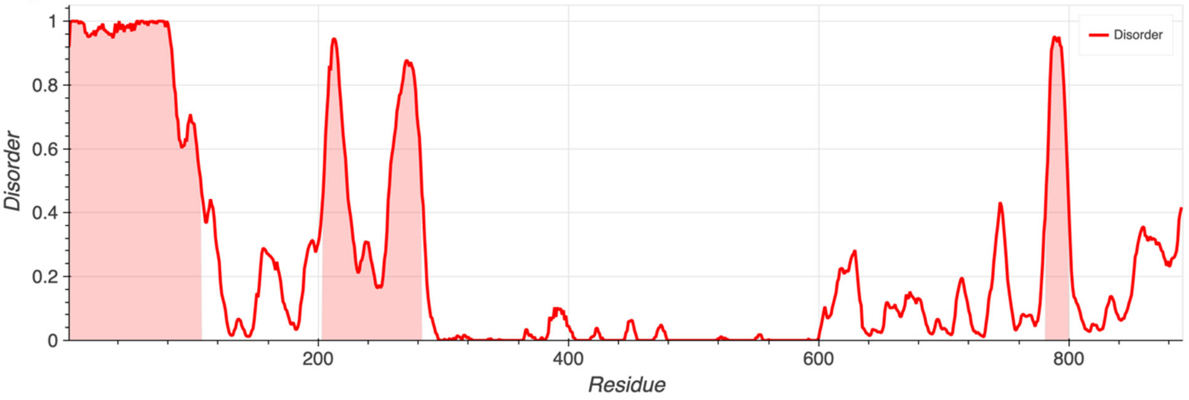

Fig. S2b.

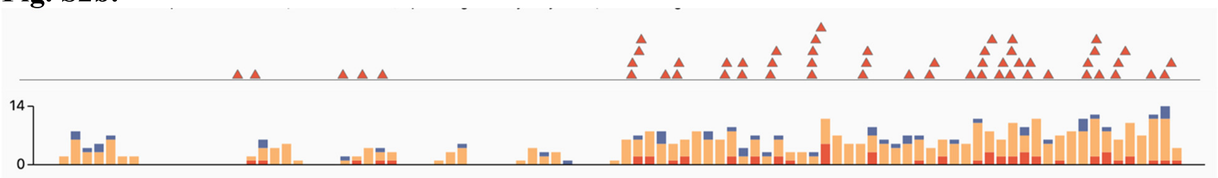

Figure S2. (a) Metapredict generated plots of intrinsically disordered regions as a function of amino acid residue **(b)** overlay of pathogenic missense variants (top panel) and total missense variants (bottom panel) for WFS1.

Fig. S3a.

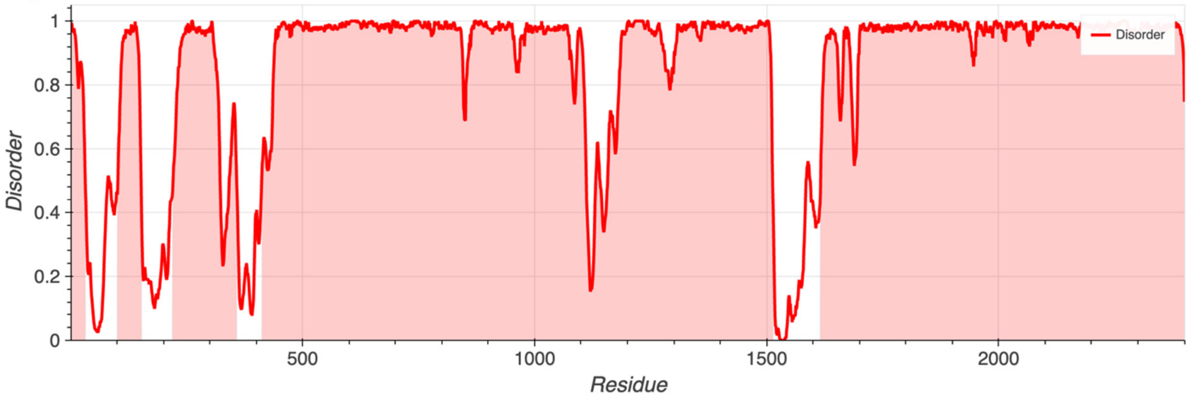

Fig. S3b.

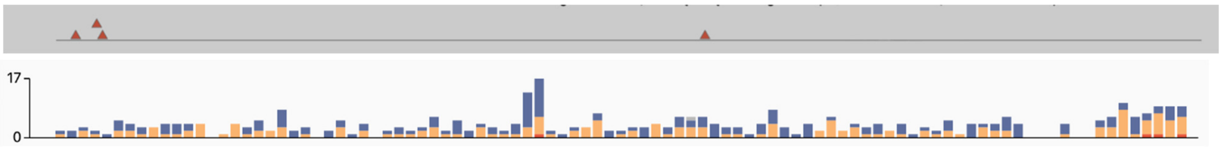

Figure S3. (a) Metapredict generated plots of intrinsically disordered regions as a function of amino acid residue **(b)** overlay of pathogenic missense variants (top panel) and total missense variants (bottom panel) for RP1L1.

Fig. S4a.

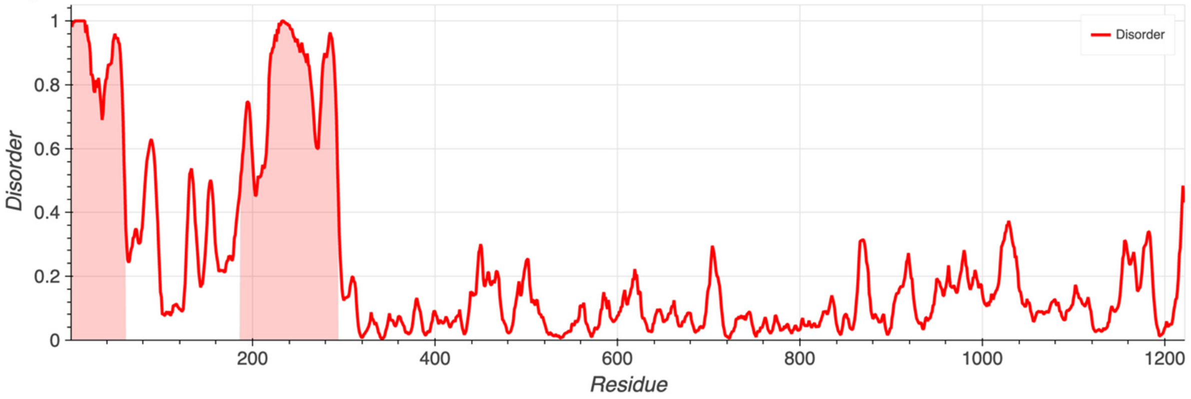

Fig. S4b.

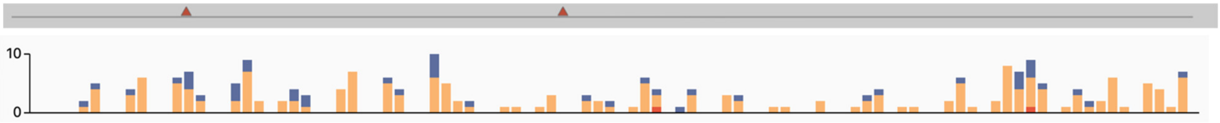

Figure S4. (a) Metapredict generated plots of intrinsically disordered regions as a function of amino acid residue **(b)** overlay of pathogenic missense variants (top panel) and total missense variants (bottom panel) for ADAMTS18.

Fig. S5a.

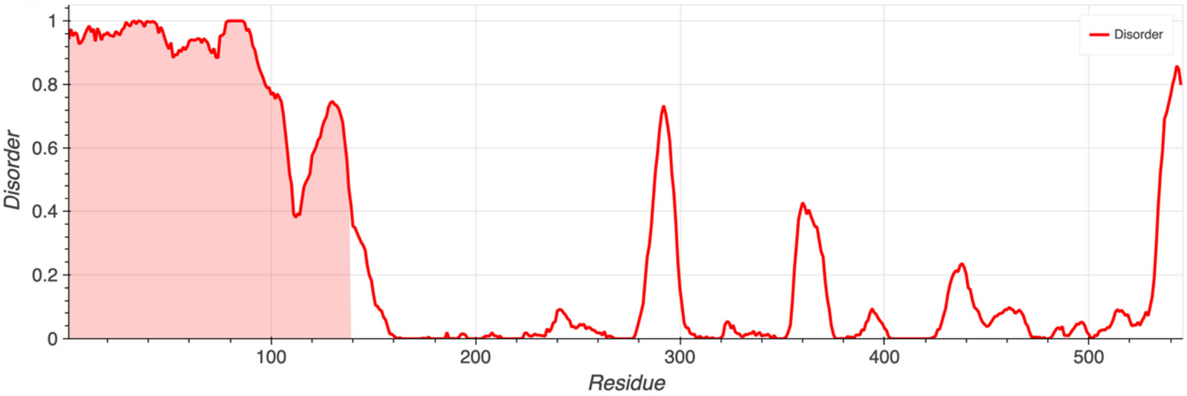

Fig. S5b.

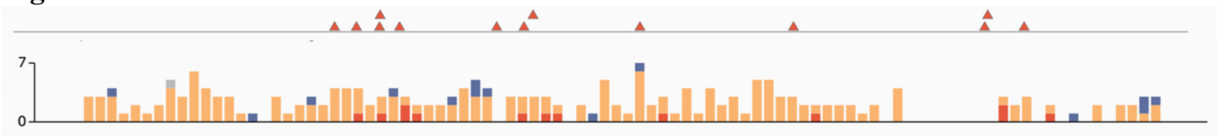

Figure S5. (a) Metapredict generated plots of intrinsically disordered regions as a function of amino acid residue **(b)** overlay of pathogenic missense variants (top panel) and total missense variants (bottom panel) for KCNV2.

Fig. S6a.

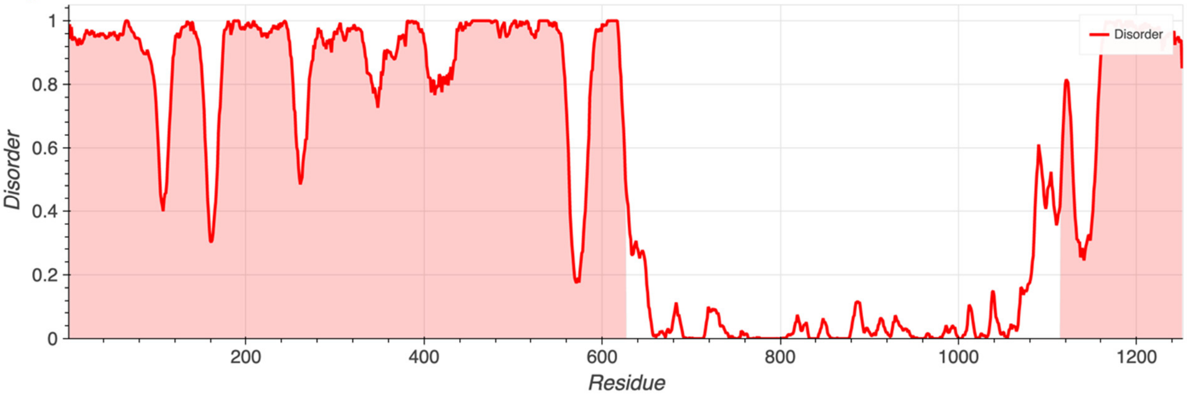

Fig. S6b.

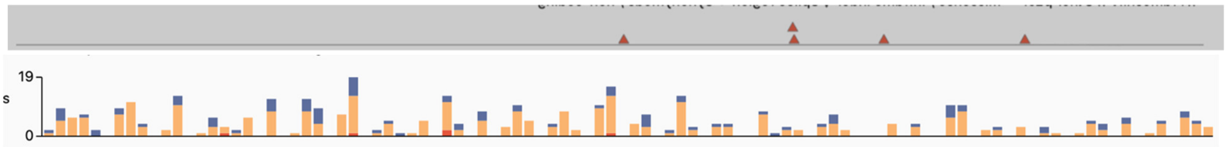

Figure S6. (a) Metapredict generated plots of intrinsically disordered regions as a function of amino acid residue **(b)** overlay of pathogenic missense variants (top panel) and total missense variants (bottom panel) for CNGB1.

Fig. S7a.

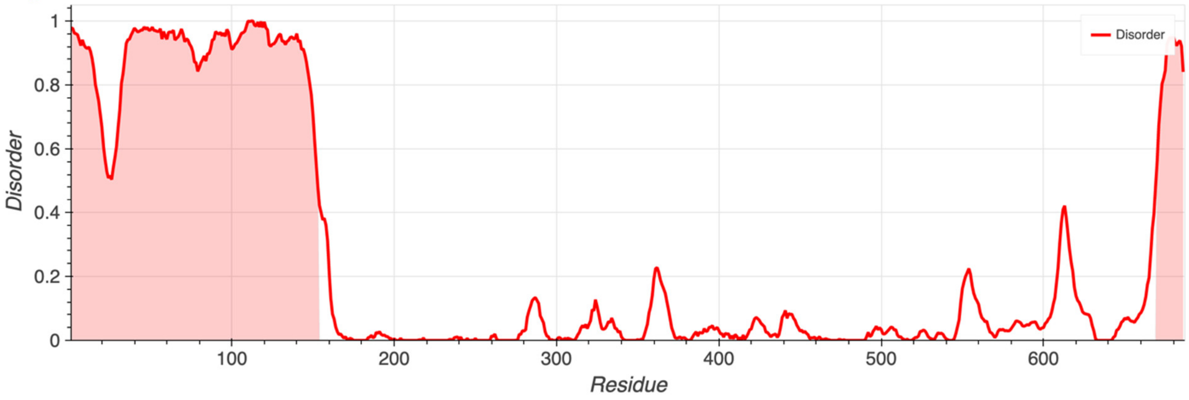

Fig. S7b.

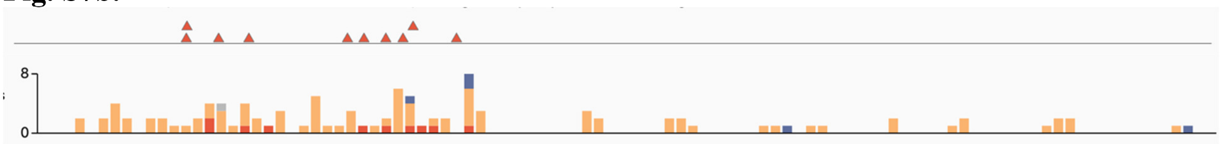

Figure S7. (a) Metapredict generated plots of intrinsically disordered regions as a function of amino acid residue **(b)** overlay of pathogenic missense variants (top panel) and total missense variants (bottom panel) for CNGA1.

Fig. S8a.

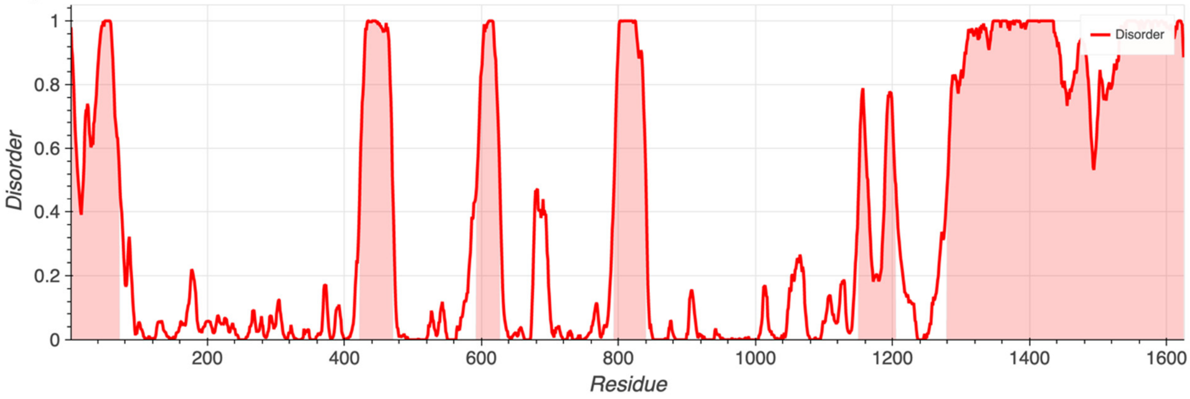

Fig S8b.

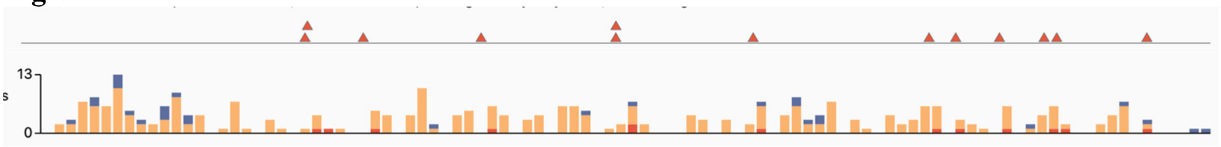

Figure S8. (a) Metapredict generated plots of intrinsically disordered regions as a function of amino acid residue **(b)** overlay of pathogenic missense variants (top panel) and total missense variants (bottom panel) for TRPM1.

Fig. S9a.

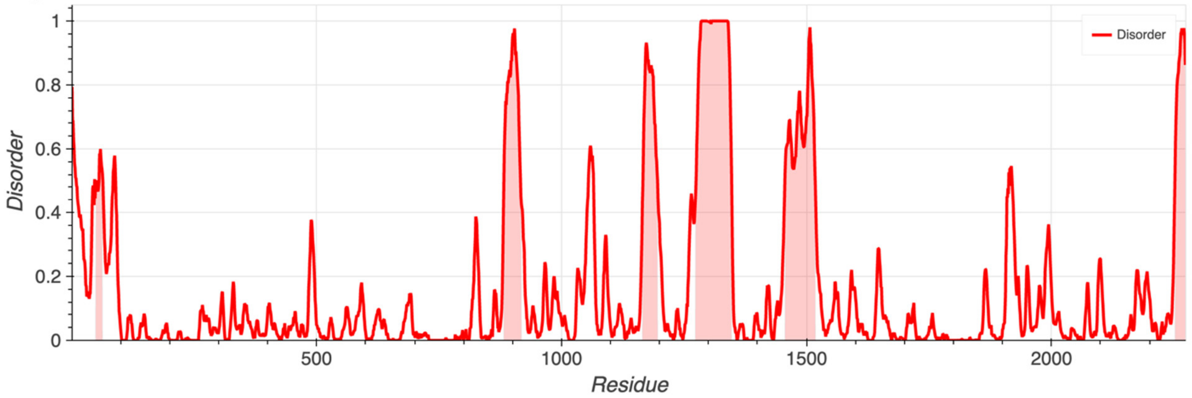

Fig. S9b.

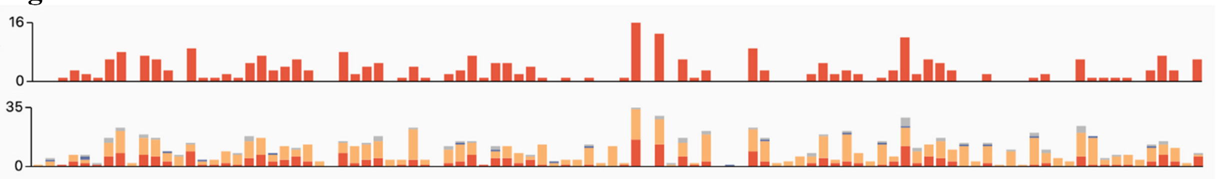

Figure S9. (a) Metapredict generated plots of intrinsically disordered regions as a function of amino acid residue **(b)** overlay of pathogenic missense variants (top panel) and total missense variants (bottom panel) for ABCA4.

Fig. S10a.

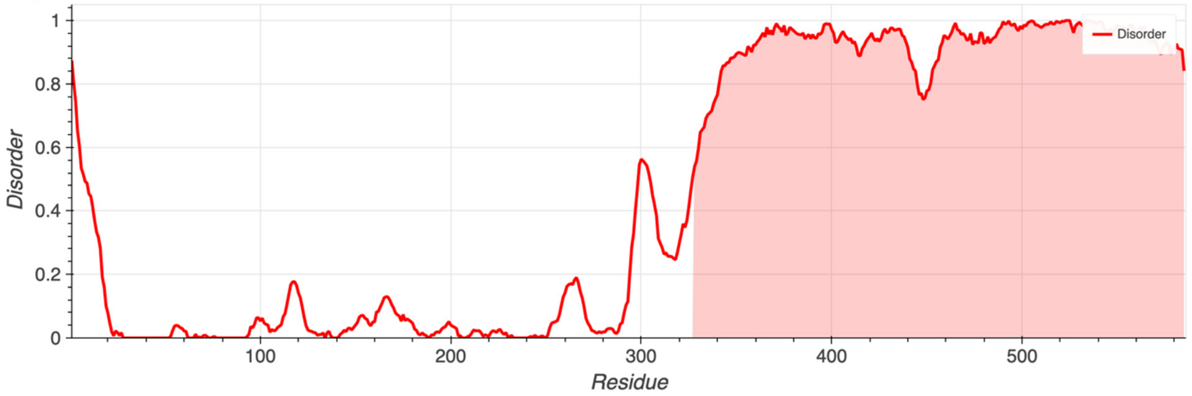

Fig. S10b.

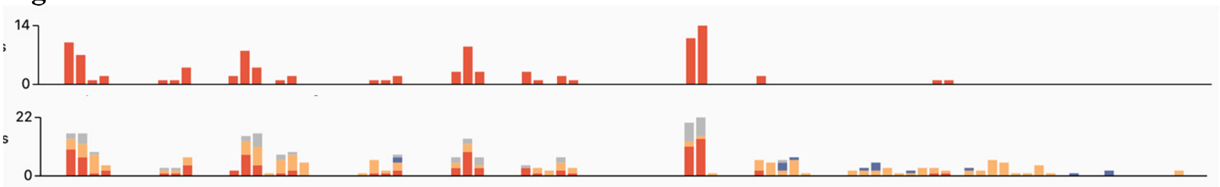

Figure S10. (a) Metapredict generated plots of intrinsically disordered regions as a function of amino acid residue **(b)** overlay of pathogenic missense variants (top panel) and total missense variants (bottom panel) for BEST1

Fig. S11a.

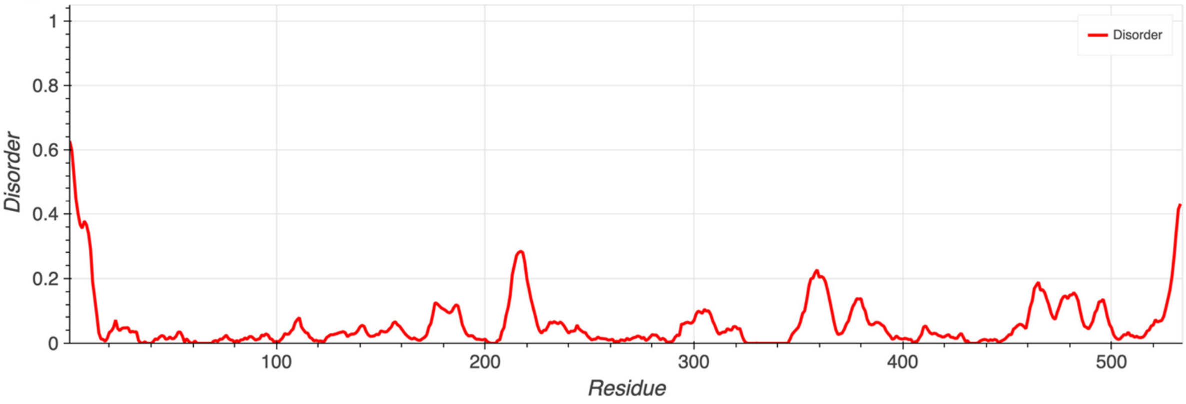

Fig. S11b.

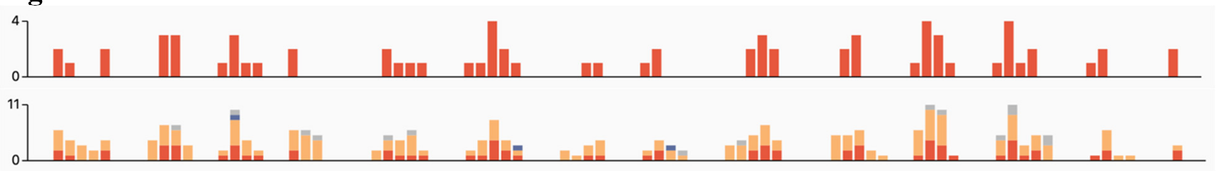

Figure S11. (a) Metapredict generated plots of intrinsically disordered regions as a function of amino acid residue **(b)** overlay of pathogenic missense variants (top panel) and total missense variants (bottom panel) for RPE65.

Fig. S12a.

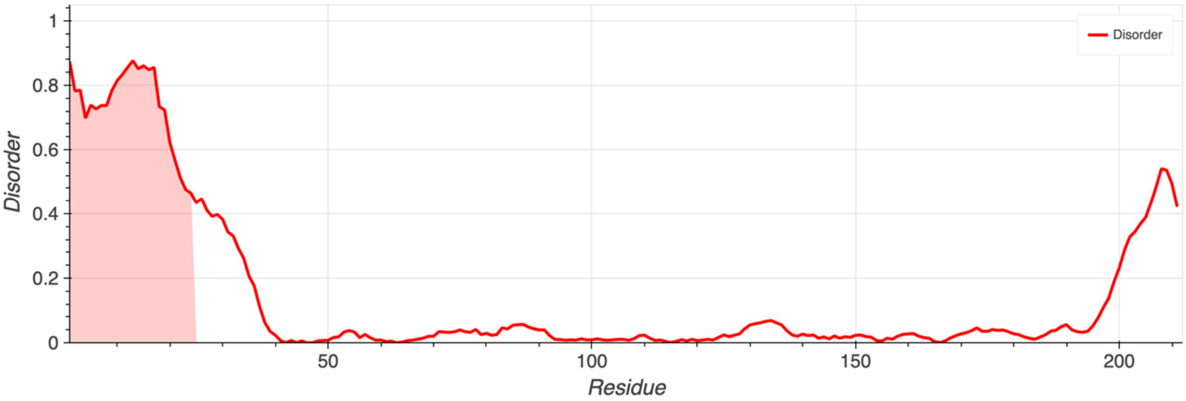

Fig. S12b.

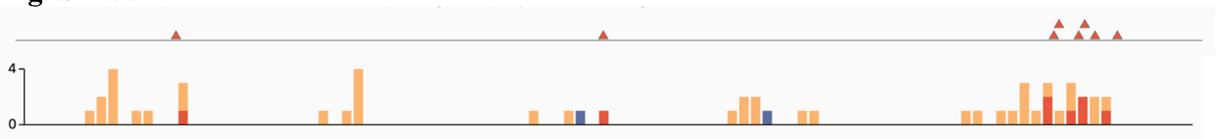

Figure 12. (a) Metapredict generated plots of intrinsically disordered regions as a function of amino acid residue **(b)** overlay of pathogenic missense variants (top panel) and total missense variants (bottom panel) for TIMP3.
